# Adaptation Modulates Spike-Phase Coupling Tuning Curve in the Rat Primary Auditory Cortex

**DOI:** 10.3389/fnsys.2020.00055

**Published:** 2020-08-03

**Authors:** Mohammad Zarei, Mohsen Parto Dezfouli, Mehran Jahed, Mohammad Reza Daliri

**Affiliations:** ^1^School of Cognitive Sciences (SCS), Institute for Research in Fundamental Sciences (IPM), Tehran, Iran; ^2^School of Electrical Engineering, Sharif University of Technology (SUT), Tehran, Iran; ^3^Neuroscience and Neuroengineering Research Laboratory, Department of Biomedical Engineering, School of Electrical Engineering, Iran University of Science and Technology (IUST), Tehran, Iran

**Keywords:** neural adaptation, spike-LFP coupling, auditory cortex, sensory coding, tuning curve

## Abstract

Adaptation is an important mechanism that causes a decrease in the neural response both in terms of local field potentials (LFP) and spiking activity. We previously showed this reduction effect in the tuning curve of the primary auditory cortex. Moreover, we revealed that a repeated stimulus reduces the neural response in terms of spike-phase coupling (SPC). In the current study, we examined the effect of adaptation on the SPC tuning curve. To this end, employing the phase-locking value (PLV) method, we estimated the spike-LFP coupling. The data was obtained by a simultaneous recording from four single-electrodes in the primary auditory cortex of 15 rats. We first investigated whether the neural system may use spike-LFP phase coupling in the primary auditory cortex to encode sensory information. Secondly, we investigated the effect of adaptation on this potential SPC tuning. Our data showed that the coupling between spikes’ times and the LFP phase in beta oscillations represents sensory information (different stimulus frequencies), with an inverted bell-shaped tuning curve. Furthermore, we showed that adaptation to a specific frequency modulates SPC tuning curve of the adapter and its neighboring frequencies. These findings could be useful for interpretation of feature representation in terms of SPC and the underlying neural mechanism of adaptation.

## Introduction

Neural adaptation is a brain mechanism that observed in various sensory systems of mammals and amphibians, including the visual (Müller, 1999; Kayser et al., 2009), auditory (Bibikov, 1977; Dean et al., 2005; Anderson et al., 2009; Malmierca et al., 2009; Hagan et al., 2012; Parto Dezfouli and Daliri, 2015), and somatosensory (Katz et al., 2006; Adibi et al., 2013, 2014; Ahmadi et al., 2019) systems. Earlier studies have reported an interesting adaptation behavior in certain neurons, including in the auditory system, so-called as stimulus-specific adaptation (SSA) (Ulanovsky et al., 2003, 2004; Nelken and Ulanovsky, 2007; Szymanski et al., 2009; Ayala and Malmierca, 2012; Khouri and Nelken, 2015; Parto Dezfouli and Daliri, 2015), here on referred to as “Adaptation.” SSA leads to a significant decline in the corresponding responses of frequent stimuli. For example, an oddball sound releases a stronger response compared to the common one. Initially, researches believed that this phenomenon was related to cortical processes, but additional evidence revealed similar behavior in other subcortical routes, such as medial geniculate body (MGB) (Anderson et al., 2009), and Inferior Colliculus (IC) (Ayala and Malmierca, 2012).

Adaptation decreases the neuronal activities in the sensory areas and leads to a system that is not disturbed in exposure to frequent stimuli (Dean et al., 2005; Adibi et al., 2013). Also, adaptation changes the system sensitivity during the action of the stimulus (Chen et al., 2010). To suppress the attention to repeated stimuli, the adaptation mechanism alters several neural properties. For instance, it helps to better detect deviance by increasing the neural sensitivity related to an unexpected change (Ulanovsky et al., 2003). In the auditory system, different parameters of a stimulus such as intensity, tone frequency distance, and Inter-Stimulus Interval is affected by adaptation. Additionally, it has been shown that presenting an audio sequence in a random pattern significantly affects the neural responses (Yaron et al., 2012).

Synchronous neural activity, alongside neural desynchrony, has been vastly studied in neuroscience, with implications for sensory information encoding and decoding, memory, attention, adaptation, and high cognitive process (Eckhorn and Obermueller, 1993; Galarreta and Hestrin, 2001; Uhlhaas et al., 2009; Pipa and Munk, 2011; van Wijk et al., 2012; Mendoza-Halliday et al., 2014; Li et al., 2015; Merrikhi et al., 2017, 2018). Local field potentials (LFPs), as the low-frequency part of neural signal, reflect the common synaptic activity of a population of neighboring neurons (Buzsáki and Draguhn, 2004; Buzsáki et al., 2012; Jansen et al., 2015), while spikes are short-timed high-frequency content signals reflecting more individual activity. Neuronal synchronization can be addressed by temporally relating spiking activities to the background oscillations of LFPs (Salinas and Sejnowski, 2001; Pikovsky et al., 2002; Tiesinga et al., 2008; Fries, 2009). This relationship has observed in various cognitive functions and within different brain regions, including the prefrontal cortex, cortical area, and hippocampus (Siegel et al., 2009; Cutsuridis and Hasselmo, 2011). The relationship further reveals information on the neuronal synchronization in each frequency band. For instance, the relation between spikes and its corresponding theta fluctuations of LFP in hippocampus neurons reflects spatial memory information (Cutsuridis and Hasselmo, 2011). Also, spike-LFP Phase Coupling (SPC) can provide information about cell type and firing rate, and avoids volume conduction complications (Canolty et al., 2010; Hoerzer et al., 2010; Womelsdorf et al., 2010; Hansen and Dragoi, 2011; Vinck et al., 2012; Xu et al., 2013; Herreras, 2016). There are different measures for estimating spike-LFP synchronization, including coherence coefficient and cross-correlation (Carter et al., 1973; Carter, 1987; Zeitler et al., 2006; Srinath and Ray, 2014), spike-triggered correlation matrix synchronization (SCMS) (Li et al., 2016), phase-locking value (PLV) (Lachaux et al., 1999), and spike field coherence (SFC) (Fries et al., 2001, 2002; Curtis et al., 2009; Grasse and Moxon, 2010; Hagan et al., 2012). PLV is considered as one of the fundamental approaches to estimate synchronization. However, this measure is highly biased and dependent on spike rates. Accordingly, before using PLV method, spikes should be matched at a specified rate. Therefore, using a scheme, the spikes equalized to a specific threshold *T*; trials with a number of spikes below *T* are discarded, and those with spike rate more than *T*, are randomly equalized down to the number of *T*.

It is known that the sensory information, namely various stimuli tuning curves, represent by spiking activity or LFPs. The power variation of LFPs could reflect various features of stimuli like tone frequency, orientation, motion, etc (Siegel and König, 2003; Henrie and Shapley, 2005; Ray et al., 2008; Kayser et al., 2009; Ince et al., 2012; García-Rosales et al., 2018a, b). Neuronal spiking activity is also able to reveal stimuli information. Relating these two signals (spike and LFP) provides a comprehensive explanation about the neural activities (Quiroga and Panzeri, 2009; Perge et al., 2014). Considering the information of spike times together with the LFP phase reveals different features in various cognitive functions (Lachaux et al., 1999; Pesaran et al., 2002, 2008; Ray and Maunsell, 2010; Vinck et al., 2010; Li et al., 2017). In fact, the coupling of spikes of single neurons to the phase of LFPs (spike-LFP phase coupling) has been a useful measure to decode the sensory information and behaviors in low and high-frequency bands (Mehring et al., 2003; Mollazadeh et al., 2009). Furthermore, a recent study revealed the spike-LFP coupling within and between areas, i.e., spikes-LFP relation in V1, spikes-LFP relation in V4, and the relation between spikes of V4 and LFP of V1 (Li et al., 2019).

We previously showed that SSA suppresses the coupling of spikes to the beta phase of LFP oscillations (Parto Dezfouli et al., 2019). Here, we sought to investigate the effect of SSA on neighboring frequencies in terms of SPC responses. To this end, we first assessed whether the spike-LFP phase coupling has a tuned response for encoding sensory information, here in the rat primary auditory cortex. In other words, we examined a potential link between the spike-LFP signals and stimuli in terms of the tone frequency tuning curves (frequency selectivity). Notably, in this study, we used the term “tuning curve” for frequency tuning curve. Second, we explored how this adaptation would alter the SPC response.

## Materials and Methods

The surgery procedure, experimental recording, and data preprocessing are described in Parto Dezfouli and Daliri (2015). Further details, employed in the current study, are provided below.

### Recording

The data was collected from the primary auditory cortex of 15 anesthetized rats. The adult Wistar rates weight ranges between 250 and 350 g. The recording was done using tungsten electrodes (FHC, 5M, United States). The parallel electrodes (tip diameter of ∼5–10 um) were placed with 200 um distance from each other. The recording electrodes were inserted into the desired location by a Microdrive (SM-21, Narishige, Japan). Multi-unit activity (MUA) and LFP were collected over A1 area with 10 kHz sampling rate (recording system: USB-ME64-PGA; Multichannel System, Germany). Through the experiment using “MCRack” software, the data was visualized online. We used an eight-channel miniature preamplifier to pre-amplify the raw signals. Next, a band-pass filtered from 1 to 5 kHz was applied to them and amplified again with a gain of 1000. Finally, the recorded data was stored for subsequent offline analyses.

### Experimental Paradigm

**FIGURE 1:**
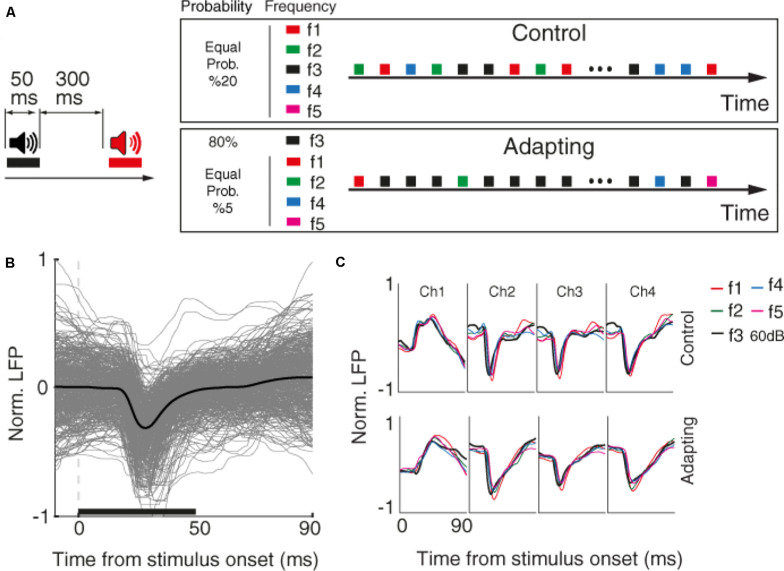
Behavioral task and raw data. **(A)** Timeline of the stimulus presentation in two control and adapting sequences. Left: pure tone stimuli with the duration of 50 and 300 ms inter-stimulus interval were presented. Right: stimuli were presented in two adapting and control sequences. Within the control sequence, stimuli were presented with an identical probability. Within the adapting sequence, the same combinations as the control sequence were presented such that the adapter and rest frequencies were presented with the likelihood of 80 and 20% of the whole sequence, respectively. **(B)** Raw LFP for a sample recording site. The *x*-axis shows the time from stimulus onset, and the *y*-axis is normalized LFP amplitude. Gray lines show LFP trace of different trials and black line denotes the average response. The horizontal bar shows the stimulus period. **(C)** Average raw LFPs of five frequencies of pure tones at 60 dB SPL level for a sample recording site (site 14) during the time following stimulus onset, in control (top panel), and adapting (bottom panel) conditions.

First, we characterized the selective neuron by presenting 300 ms broad-band noise bursts with 500 ms interval between them. Next, for each selected recording site, we identified the characteristic frequency and four frequencies around it (in the range of 200 Hz–20 kHz). These five desired tone frequencies (namely f1–f5) were presented in four intensities 40, 50, 60, and 70 dB SPL in a quasi-random pattern. Each tone was presented for 50 ms duration with a 300 ms inter-stimulus interval (Figure 1A).

The main task consists of two control and adapting sequences. In the control sequence, 20 selected combinations were uniformly presented (30 trials of each combination). In the adapting sequence, a similar procedure was conducted but with different likelihood of stimuli presentation. In this sequence, the characteristic frequency (f3) in the level of 60 dB SPL (as the adaptor) was presented for 80% of all sequence likelihood. Accordingly, each frequency of pure tones was presented with the same probability of 20% in the control sequence, while that probability was 80% for the adaptor and 5% for neighboring frequencies in the adapting sequence.

### Data Analysis

All preprocessing and analyses were implemented in MATLAB 2016b (Mathworks, Natick, MA, United States). LFP signals, were filtered between the ranges of 1–300 Hz. Subsequently, using 300–3000 Hz band-pass filtered MUAs were extracted. Next, we employed a threshold method to detect spike times. The threshold may be set based on the standard deviation (SD) of the whole trace, namely as twice the SD, as considered in this study (Pouzat et al., 2002). The resulting spike trains were smoothed using a 10 ms Hamming window and aligning to the stimulus onset. For LFPs, after 1–300 Hz filtering, the baseline correction was applied to each trace (Parto Dezfouli et al., 2014). Subsequently, the preprocessed data were divided to different canonical frequency bands using band-pass filters and non-causal finite impulse response (FIR) filter. We used the time between 0 and 100 ms after the stimulus onset for the main analysis.

### SPC Based on the PLV Method

Here, we utilized PLV to quantify SPC (Lachaux et al., 1999). PLV method calculates the power of dependability or linking of LFP phases in spike times, by computing the angular summation between spikes to beta range LFP fluctuations. We used the following equation:

(1) $$M=\frac{1}{N}\,\left|\sum_{n=1}^{N}e\,x\,p\,\left(j\,\phi_{n}\right)\right|$$

where N shows the number of spikes, ϕ_n_is related to the instantaneous phase (here in the beta-band) at the time of nth spike which is determined by Hilbert transform, and *exp*⁡(jϕ_n_)is the complex exponential function of ϕ_n_. The amplitude of vector M (|M|) indicates the SPC power and its angle (∠M) shows the phases of LFPs in the time of spikes occurrence. A larger value for vector M indicates that the occurrence of spikes are more likely to a specific phase of LFP, and the smaller value is related to distributed spikes across different phases. PLV alters between 0 and 1.

As noted, the dependency of SPC value to the spike numbers is considered as one limitation of SPC estimation. For example, two neurons with various firing rates, it is distinctly possible that the neuron with a greater firing rate results in a lower SPC. This problem was targeted to address in previous studies on SPC (Vinck et al., 2012; Zarei et al., 2018).

In this study, to calculate the SPC by PLV, an equalizing strategy was used in order to find the spike counts based on a threshold. Here we reach an optimal compromise between spike rates and trial numbers using an optimal thresholding scheme (Zarei et al., 2018). Accordingly, the trials with spike rates greater than the threshold were equalized to that threshold (by randomly removing), and the trials whose number of spikes were less the threshold, were removed.

To overcome this problem, we matched the firing rates in the control and adapting conditions. Therefore, after finding a threshold *T* for the mean firing rates, trials with firing rates less than the *T* were removed and spikes in trials with firing rates greater than the *T* were reduced to the *T* value. Especially, in order to produce normalized LFP signals, the LFP strength for each neuron was standardized by deducting the average and dividing the result by the SD.

In order to quantify the adaptation effect, we computed the difference between the firing-rate/SPC strength of control and adapting conditions. We measured the adaptation changes using Adaptation Index (AI) in an analysis similar to SSA index (SI) that was employed in earlier studies (Ulanovsky et al., 2003, 2004), and is defined as,

(2) $$A\,I=\frac{C\,\left(f\,i\right)-A\,\left(f\,i\right)}{C\,\left(f\,i\right)+A\,\left(f\,i\right)}$$

where the parameters C(fi) and A(fi) are the response (firing-rate/SPC) strength related to the frequency fi in the control and adapting sequences, respectively. The AI denotes the difference between control and adapting sequences within each stimulus tone frequency. Therefore, the AI value shows the difference between control and adapting responses (firing-rate/SPC).

### Fitting Model

In many signal processing subjects fitting Gaussian functions to neural data is very essential, that a Gaussian function is of the following form:

$$y={\text{Ae}}^{{}^{-{\left(x-\mu \right)}^{2}\,/\,2\,\sigma^{2}}}$$

This function can be mapped with a symmetrical bell-shaped curve positioned at the place x = μ, with A being the altitude of the peak and σ utilizing its width.

### Quantification and Statistical Analyses

#### Wilcoxon Rank-Sum

We employed the Wilcoxon rank-sum test for statistical assessment of the firing rate and SPC between the characteristic frequency and its neighboring frequencies across neurons (Figures 4A,B).

#### Standard Error of Mean (SEM) and Standard Deviation (SD)

Standard error of mean (SEM) and SD were used to convey variability through different measures, where SEM exemplify uncertainty in the assessment of the mean and SD illustrates a scattering of the data from the mean (Figures 3A, 4A,B, 5).

#### Correlation

In this study, the Pearson’s correlation was used between the mean firing rate and SPC strength (Figure 4B). Pearson’s correlation is a statistical measure that quantities linear correlation between two variables. It assumes a value between (−1 and +1), where −1 depicts a negative correlation, 0 shows no correlation, and −1 represents a positive correlation.

## Results

We investigated sensory information coding in terms of SPC tuning curve and then explored how adaptation could alter this potential SPC-based tuning curve. To this end, we used data of an auditory task consisting of two usual and adapted conditions. Figure 1A depicts the timeline and phases of the auditory task made up of two sequences; control and adapting. Pure tones in 20 arrangements of five frequencies and four intensity levels were employed in the experiment. In the control sequence, stimuli were randomly presented with an equal likelihood of 5% for each combination. The adapting sequence is constituted of the same stimuli but with different probabilities of stimuli presentation. In this sequence, an adapter (characteristic frequency, f3, at 60 dB SPL intensity) was presented with 80% probability of the whole sequence and other four frequencies were occupied the rest 20% of the sequence. During the experiment, neural data (LFP and MUA) were collected from 96 sites over the primary auditory cortex (A1). Raw LFPs of a sample recording site and the average of these raw LFPs for each of the five tone frequencies are shown in Figures 1B,C, respectively.

**FIGURE 2:**
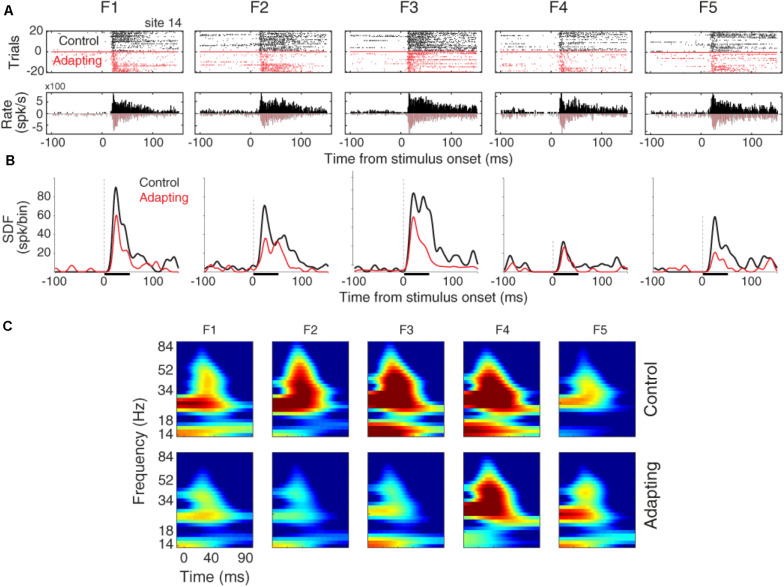
Adaptation impacts on the neuronal spiking activity and LFP responses. **(A)** Raster plot and peristimulus time histogram (PSTH) of the adapter (tone frequency f3) and its neighboring frequencies at 60 dB SPL intensity for a sample recording site (site 14) in control (black) and adapting (red) sequences. **(B)** Comparison of spike density function (SDF) between control (black) and adapting (red) sequences for the five respective frequencies of pure tones at 60 dB SPL intensity. **(C)** Time-frequency representation of the LFP power for different stimuli in a sample recording site (site 14). Color bars show the mean normalized LFP power, as a function of time (*x*-axis) and LFP frequency (*y*-axis) in the control (top panels) and adaptation (bottom panels) sequences.

The raster plots and peristimulus time histograms of the five desired tone frequencies related to a sample recording site are shown for the control and adapting conditions, separately (Figure 2A). Consistent with previous findings (Ulanovsky et al., 2003; Nelken and Ulanovsky, 2007), spiking activity shows a suppression due to the adapting effect (Figure 2A). To have a better estimation of neural responses, we convolved a Gaussian Kernel (with σ = 10) with the spikes (Hill et al., 2015). This resulted in a continuous probability density function as illustrated in Figure 2B. Figure 2C shows the time-frequency representation of the adapter (f3) and the neighboring frequencies (f1, f2, f4, f5), for site 14. Consistent with Figure 2A, the adaptation caused suppression in the LFP power of the adapter and the neighboring frequencies. This adaptation effect is shown to be stronger in the characteristic frequency (f3), as compared to the neighboring frequencies.

### Sensory Representation Based on Spike-LFP Coupling

**FIGURE 3:**
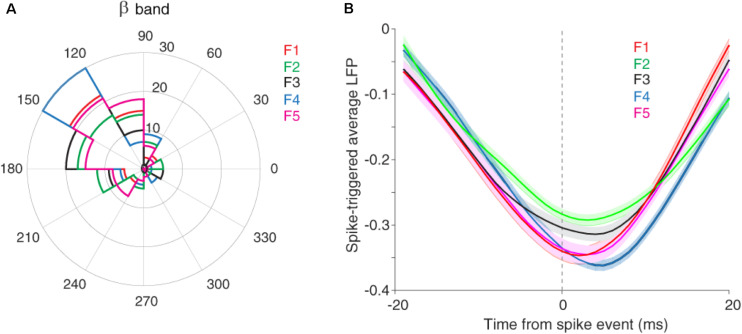
The preferred LFP phase. **(A)** The LFP phase histograms of different stimuli (f1–f5) within the beta range (13–30 Hz), show approximately identical locking phase mean of ∼140° for five desired tone frequencies. **(B)** STA calculated across all recording sites and for each five desired tone frequencies separately.

It has been shown that the neuronal spiking activity and cortical LFP are attenuated due to adaptation (Taaseh et al., 2011; Parto Dezfouli and Daliri, 2015). Recently, we showed that adaptation decreases the spike to LFP phase coupling within beta range but not in other frequency bands (Parto Dezfouli et al., 2019). To this end, we divided LFP to six canonical frequency bands, namely delta, theta, alpha, beta, low and high gamma. Results indicated a significant difference in the SPC values between control and adaptation conditions within the beta range, but not in other bands (Parto Dezfouli et al., 2019). Therefore, here, we focused our analyses within the beta range (13–30 Hz). Based on our previous findings, the primary auditory system may further use this SPC within the beta range to encode sensory information. We considered the phase with dominante occurrence of spikes as the preferred, as the preferred phase (α) and the phase with 180° distance from it (180−α) as the anti-preferred phase. The preferred phase was identified by calculating the histogram of LFP phases in spike times. The LFP phase histograms (in the beta range) for different stimuli, namely conditions of five desired tone frequencies (f1–f5) were shown in Figure 3A. As described, the LFP phase distributions at different stimulus frequencies differ significantly from the uniform distribution. The different locking phases for the five desired tone frequencies (f1–f5) amount to almost identical locking phase means of about 140°, with no significant statistical difference (*p* = 0.3, *t*-test). This shows that auditory neurons tend to fire more likely in a specific phase within the beta range (13–30 Hz) of LFPs. This effect is observed in both adapting or control conditions, and independent of different stimulus frequencies.

We also estimated the preferred LFP phase using the spike-triggered average (STA) method. For this purpose, after detecting the spike times, the LFPs within a window (±20 ms) around spike times were averaged. Figure 3B shows that the coupling strength, defined as the difference between the peak and trough of the STA curve, is different in the certain phase (phase of the coupling) for various stimuli (f1, f5). In other words, the strength of coupling in the primary auditory cortex neurons encodes sensory information. Moreover, Figure 3B indicates the falling phase (∼160°) as the preferred phase in which spikes are coupled to (consistent with Figure 3A). In the following, we examine this discrimination of sensory information for different stimuli in the format of a tuning curve (TC).

### SPC Follows an Inverted Bell-Shaped Tuning Curve

To investigate if the coupling between spikes and LFP phase encodes sensory information in the rat primary auditory cortex, we measured the coupling between spikes and the phase of beta-frequency oscillations of LFP as a function of different stimulus frequencies.

**FIGURE 4:**
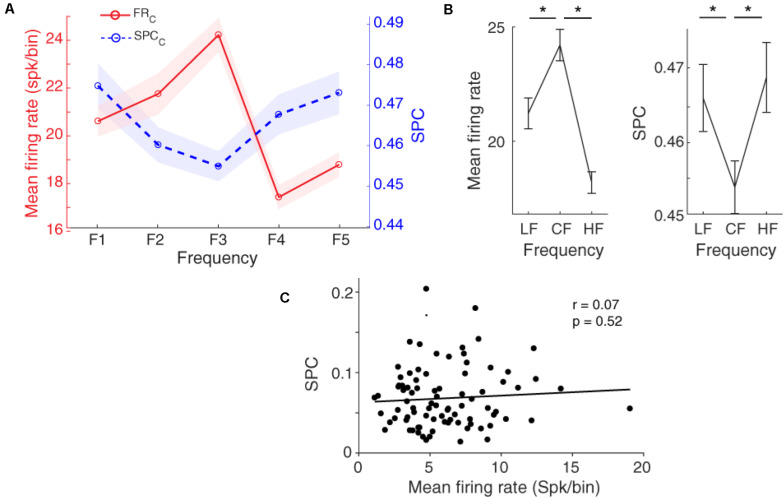
Spike-phase coupling (SPC) tuning curve. **(A)** The firing rate and SPC strength relative to different stimulus frequencies averaged for the population of neurons. The *x*-axis shows different frequencies and the *y*-axes represent the normalized spike rate (red solid curve) and SPC strength (blue dashed curve). Resembling the firing rate with a bell-shaped tuning curve, the SPC strength relative to different stimuli follows an inverted bell-shaped tuning curve. Shaded areas show SEM. **(B)** Statistical comparison of the firing rate (left panel) and SPC (right panel) between the characteristic frequency (CF) and its neighboring frequencies (LF, HF). Stars show a significant difference (Wilcoxon rank-sum test, *p* < 0.05). LF is F1 and F2 responses, and HF is related to F4 and F5 responses. **(C)** The correlation between mean firing rate and mean SPC across different recording neurons. No significant correlation between them was observed (Pearson’s correlation, *p* > 0.05).

Figure 4A shows that the locking of spikes to the LFP phase follows a tuning curve based on the different frequencies of the presented stimulus. Statistical comparison between the characteristic frequency and its lower and higher neighboring frequencies (LF and HF, respectively) shows a significant difference between them (Figure 4B, Wilcoxon rank-sum test, *p* < 0.05), averaged across neurons (*n* = 96) within the 13–30 Hz band. The SPC strength follows a shape of an inverted bell tuning curve relative to the different tone stimulus frequencies (using fitting model Piecewise linear interpolation). This tuning curve is inverted compared to the tuning based on the spike rate. That is, the least SPC occurs for the characteristic frequency as determined based on the spike rate, while the largest spike-LFP phase coupling is induced by the neighboring frequencies.

To evaluate the relation of SPC (based on LFP phase at the beta range) and spike rate in the tuning curve, we computed the correlation between mean spike rate and mean SPC for characteristic frequency and neighboring frequencies in all individual neurons. Results show no significant correlation between the mean firing rate and SPC strength (Figure 4C; Pearson correlation, *r* = 0.07, *p* = 0.52). It shows that SPC is mechanistically independent of the spike rate.

### Adaptation Modulates SPC Tuning Curve

**FIGURE 5:**
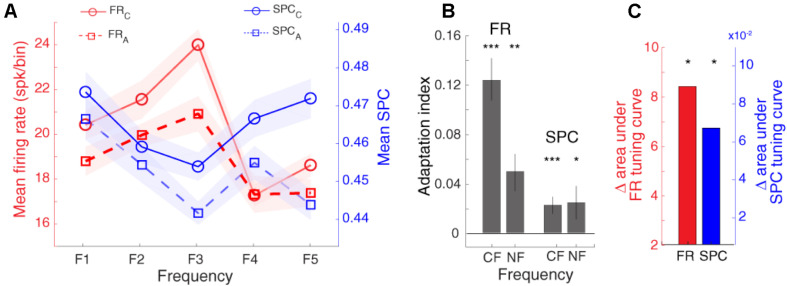
Adaptation modulates spike-phase coupling (SPC) tuning curve. **(A)** The firing rate and SPC strength relative to different stimulus frequencies are averaged for the population of neurons. The *x*-axis shows different frequencies and *y*-axes represent the normalized spike rate (solid-curve) and SPC strength (dashed-curve). The spike rate tuning curve and the strength of SPC tuning curve were suppressed in the adapting condition (blue) compared to the control condition (red). **(B)** Statistical comparison of adaptation effect. Adaptation Index (*y*-axis) that was achieved from the firing rate, and SPC analyses. The suppression due to adaption is significant in the characteristic frequency (CF) and its neighboring frequencies (NF; f1, f2, f4, f5) (*t*-test; *p < 0.05, **p < 0.01, ***p < 0.001). This effect is observed both based on firing rate and SPC. **(C)** Δ area under tuning curve. The red and blue bars show the difference between the area under solid and dashed curves in firing rate and SPC tuning curves, respectively.

As expected, Figure 5 shows that the spike rate tuning curve (SR-TC) is attenuated in the adapting condition in comparison to the control condition (Taaseh et al., 2011; Parto Dezfouli and Daliri, 2015). Furthermore, as mentioned in Figure 4A, SPC strength relative to different stimuli follows a tuning curve (SPC-TC) across neurons. Importantly, adaptation modulates this SPC tuning curve across sites, in both control (blue dashed line), and adapting (red dashed-line) sequences (Figure 5). The SPC strength was decreased in the adapting sequence compared to the control sequence. This index quantifies the difference between neural responses of the two desired sequences (control and adapting). Due to the nonsymmetrical tuning curve and a non-monotonic trend of the neighboring responses, we computed AI for responses (firing-rate/SPC) of two conditions; characteristic frequency (CF) and its neighboring frequencies (NF; f1, f2, f4, f5) (Figure 5B). Also, the Δ area under tuning curve is performed using the difference between the area under control (solid) and adapting (dashed) curves in firing rate and SPC tuning curves, respectively (Figure 5C). As a result, the adaptation caused a suppression in the spiking activity and SPC of the characteristic frequency and the neighboring frequencies. In other words, adaptation shifts down both tuning curves.

Notable, the range of AI values change between −1 and +1. The positive value indicates a lower response strength for adapting compared to the control condition.

## Discussion

In this study, we found a tuning link between the sensory information and the coupling of spike times to the LFP phase. Furthermore, we revealed that the adaptation mechanism modulates this SPC tuning curve.

Stimulus-specific adaptation understood as an interesting phenomenon in the neural system, including the auditory cortical neurons, (Ulanovsky et al., 2003, 2004; Ayala and Malmierca, 2012), here on denoted to as “Adaptation.” SSA affects a major decrease in neural responses to frequent stimuli. Adaptation has a tendency to conceal neuronal activities in the sensory systems, leading to a system that is not distracted in exposure to frequent stimuli (Dean et al., 2005; Adibi et al., 2013) from the world such as light, smell, and sound. To decrease attention to frequent stimuli, the adaptation mechanism affects certain variations in neural properties.

As aforementioned, SPC indicates how spikes are harmonized in the LFPs for various functions of the brain such as attention, adaptation, perception, and maintaining information. This relationship has pointed to various brain areas, such as the visual cortex, prefrontal cortex, and the hippocampus (Siegel et al., 2009; Cutsuridis and Hasselmo, 2011). It is known that the sensory information, namely various stimuli tuning curves, represent by LFPs or spike activities (Snowden et al., 1992; Liu and Newsome, 2006; Ray and Maunsell, 2010). Importantly, relating these two signals, namely spike-LFP, provides a comprehensive explanation regarding neural activities (Quiroga and Panzeri, 2009; Perge et al., 2014). Indeed, SPC is a useful measure to decode the sensory information and behaviors in low and high-frequency bands as well as parietal and frontal cortex for alpha and beta bands (Mehring et al., 2003; Mollazadeh et al., 2009). Furthermore, LFP phase-locking was observed in the secondary auditory cortex during remote memory recall (Cambiaghi et al., 2016), where phase-locking was associated with a specific behavioral outcome.

Moreover, in line with sensory information findings, Uhlhaas et al. (2009) found that the SFC is boosted for preferred stimulus neurons in gamma band while it is reduced for the non-preferred stimulus. Belitski et al. (2010) documented the spike LFP relation may convey sensory properties in the low-frequency range and Kevan et al. revealed that the spike LFP behavior may be distinguished in reaction to various stimulus conditions (Martin and Schröder, 2016). A number of SPC quantities are studied, where the major synchronization quantities are the SCMS, pairwise phase consistency, SFC, and PLV (Lachaux et al., 1999; Grasse and Moxon, 2010; Vinck et al., 2010; Li et al., 2016). PLV technique calculates the amplitude of the average variation between spikes and LFP phases as the power of coupling. To overcome the limitation of the PLV method (bias on the number of spikes), researches that utilize this technique usually match the firing at a specific spike number using the optimal thresholding method.

The main purposes of this study were to (i) evaluate the potential of the tone frequency tuning curve (sensory information) based on SPC, and (ii) examine the effect of adaptation on this tuning curve. In a recent study, we showed that SSA reduces the SPC strength significantly in the beta range (Parto Dezfouli et al., 2019). Resembling previous procedure, in this work, we analyzed the power of SPC in terms of tuning curve for sensory information coding. Our results indicate that the SPC follows a shape of an inverted bell curve relative to the different stimulus frequencies (using fitting model Gaussian function), averaged across neurons (*n* = 96) within the 13–30 Hz band (Figure 4A). Importantly, to evaluate the relation of SPC (based on the LFP phase at the beta range) and establish that it is mechanistically independent of firing rate, the correlation computed between mean spike rate and mean SPC within the characteristic frequency and neighboring frequencies for all individual neurons. Our results show no considerable correlation between the SPC strength and mean spike rate (Figure 4B).

Previous studies revealed different tuning curves such as V shape, O shape, and bimodal peak, for the neurons in the primary auditory cortex (Sutter, 2000). Therefore, the shape of the neurons’ tuning curves is not necessarily bell-shaped or symmetrical. Therefore, instead of investigating the adaptation effect on each of the frequencies, we performed the adaptation on the whole frequencies. Namely, we computed for responses (firing-rate/SPC) of characteristic frequency (CF) and neighboring frequencies (NF; f1, f2, f4, f5) (Figure 5B). Also, the Δ area under tuning curve is performed using the difference between the area under of the solid and dashed curves in firing rate and SPC tuning curves, respectively (in terms of area under tuning curve, Figure 5C). As a result, the adaptation leads to a reduction in the spiking activity and SPC of the characteristic frequency and neighboring frequencies. Moreover, this selective function (SPC tuning) is inverted compared to the spike rate tuning. Our results illustrate that additional spikes evoked by the characteristic frequency, (compared to the neighboring frequencies) occur more frequently at the preferred compared to the anti-preferred phase of LFP. Furthermore, spikes evoked by the characteristic frequency depict a probability distribution that is less non-uniform than of the spikes induced by the neighboring frequencies. This may cause such stronger neural discrimination at the preferred compared to the anti-preferred phase of LFP, namely SPC tuning curve is inverted compared to the tuning based on the spike rate. Furthermore, we found that the adaptation modulates SPC tuning curve of the adapter and the neighboring frequencies and shift it toward lower values.

## Conclusion

This study indicates three main findings. First, the strength of SPC is selective for sensory information in the primary auditory cortex. Second, the locking of spikes to the LFP phase follows an inverted bell-shaped tuning curve relative to the different stimulus frequencies. Third, the adaptation modulates SPC tuning curve of the adapter and its neighboring frequencies.

## Data Availability Statement

The data analyzed in this study is subject to the following licenses/restrictions: The datasets analyzed in this article are not publicly available. Requests to access these datasets should be directed to MD, daliri@iust.ac.ir (upon reasonable request).

## Ethics Statement

In the current study, we aimed to investigate sensory information tuning in terms of spike-phase coupling and examine the effect of adaptation on this potential tuning curve. We conducted all procedures of this experiment in Iran Neural Technology Center (INTC) at Iran University of Science and Technology (IUST) using standards and conforming methods of the ministry of health and medical education. Animals are kept in the standard cages in animal facilities of the Neural Technology Center. All animals care, surgical procedures and experiments reviewed and approved and performed according to the protocols of the committee of Neuroscience Research Laboratory, Iran University of Science and Technology in strict accordance with the recommendations in the Guide for the Care and Use of Laboratory Animals of the National Institutes of Health. All surgeries were conducted under urethane pentobarbital anesthesia, and all protocols were considered to minimize suffering. At the final step after the experiment, the animal was sacrificed under ether anesthesia (Parto Dezfouli and Daliri, 2015).

## Author Contributions

All authors contributed to the concept of the work, drafting, and revising the manuscript. MP performed the experimental recording. MZ and MP analyzed and interpreted the data.

## Conflict of Interest

The authors declare that the research was conducted in the absence of any commercial or financial relationships that could be construed as a potential conflict of interest.
